# Closed Loop of Polyurethanes: Effect of Isocyanate Index on the Properties of Repolyols and Rebiopolyols Obtained by Glycolysis

**DOI:** 10.3390/ma18245503

**Published:** 2025-12-07

**Authors:** Maria Kurańska, Elżbieta Malewska, Julia Sędzimir, Hubert Ożóg, Aleksandra Put, Natalia Kowalik, Michał Kucała

**Affiliations:** 1 Faculty of Chemical Engineering and Technology, Cracow University of Technology, Warszawska 24, 31-155 Cracow, Poland; elzbieta.malewska@pk.edu.pl (E.M.); julia.sedzimir@student.pk.edu.pl (J.S.); hubert.ozog@student.pk.edu.pl (H.O.); natalia.kowalik18@student.pk.edu.pl (N.K.); 2 Interdisciplinary Center for Circular Economy, Cracow University of Technology, Warszawska 24, 31-155 Cracow, Poland; 3 CUT Doctoral School, Faculty of Chemical Engineering and Technology, Cracow University of Technology, Warszawska 24, 31-155 Cracow, Poland

**Keywords:** repolyol, rebiopolyol, biopolyols, polyurethane, circular economy, foams

## Abstract

This paper presents the effect of the isocyanate index of polyurethane foams on the properties of repolyols and rebiopolyols obtained through glycolysis and on the foaming process of the new polyurethane systems. An FTIR spectral analysis confirmed that as the isocyanate index decreased, the intensity of the bands’ characteristic of urethane and urea bonds also decreased, indicating a lower proportion of carbonyl groups and hard segments in the polymer structure. Simultaneously, an increase in the hydroxyl number of the repolyols and the rebiopolyols was observed, along with a decrease in their viscosity and average molar masses. Both effects are consequences of a lower degree of cross-linking in the parent foams. An analysis of the foaming process using a Foamat apparatus revealed that the viscosity and the molar mass of the repolyols and the rebiopolyols significantly affected the system’s reactivity, maximum reaction temperature, and the time required to reach it. Differences in foaming dynamics resulted in different cellular structures of the foams, their apparent density, and mechanical properties. The foams obtained from the repolyols derived from foams with a lower isocyanate index exhibited a lower degree of cross-linking and a lower strength, while the foams with the rebiopolyols tended to shrink. The intensity of the shrinkage was limited by a higher degree of cell openness. These results confirm the crucial role of the properties of repolyol and rebiopolyol in shaping the reactivity, morphology, and properties of final polyurethane foams, providing a basis for designing new, sustainable polyurethane systems.

## 1. Introduction

In 2019, the European Council introduced the European Green Deal—a framework of initiatives under the European Climate Law aimed at driving the EU’s green transition. Its key goals include reducing greenhouse gas emissions by 55% by 2030, achieving climate neutrality by 2050, increasing renewable energy use, and promoting a circular economy focused on waste reduction, reuse, and recycling [1,2,3,4]. In line with the principles of a circular economy, one of the key challenges of contemporary chemical engineering is developing effective methods for the reuse of polymeric materials, including polyurethanes (PURs). PUR ranks as the sixth most widely used polymer globally, with its production reaching approximately 24 million metric tons in 2018 and maintaining a steady annual growth rate of around 4% [5]. PURs constitute a significant group of plastics, widely used in construction, furniture, and transportation, among other industries, due to their excellent thermal insulation, mechanical and functional properties [6,7]. However, their complex, network-like chemical structure prevents traditional mechanical recycling, resulting in the majority of PUR waste ending up in landfills or incinerators [8]. Therefore, the development of chemical recycling methods that enable the recovery of useful raw materials from polyurethane waste is particularly important.

Polyurethane recycling can be achieved through various pathways: mechanical, physical, chemical, or energetic recycling [9,10]. Among these methods, chemical recycling—including processes such as glycolysis, aminolysis, alcoholysis, and hydrolysis—is the most promising, as it enables the decomposition of the cross-linked polyurethane structure and the recovery of reaction products (repolyols), which can be reused in the synthesis of new polyurethane foams [11,12]. This allows for closing the material’s life cycle, which is consistent with the principles of sustainable development and minimizes the environmental footprint.

Glycolysis is the most commonly reported method of polyurethane recycling in the literature, as it enables the effective chemical breakdown of polymer chains into reusable polyol components, allowing partial recovery of the original raw materials and supporting the principles of a circular economy [13,14]. The key factors influencing the glycolysis process and the properties of the resulting recyclate include the chemical composition of the waste PUR, the PUR-to-glycol mass or molar ratio, the type of glycol and catalyst used (basic, amine, or organometallic), and the reaction temperature [9,15,16]. Chemical recycling of rigid PUR foam waste generally yields homogeneous recyclates. In contrast, glycolysis products from flexible PUR foams often show phase separation, as the high-molar-mass polyols used in their production are typically immiscible with glycolysis products and unreacted glycol. This confirms that the chemical structure of the original PUR foam strongly affects the properties of the resulting repolyol.

The isocyanate index (INCO) of polyurethane foam is also expected to influence the properties of the resulting chemically recycled products. The isocyanate index is the ratio of the amount of isocyanate used in the foaming process to the theoretical amount calculated from the formulation, with respect to all raw materials containing groups reactive toward isocyanate groups. It represents the relationship between the actual amount of NCO groups supplied and the amount of NCO required for the stoichiometric reacting with all active groups, such as the hydroxyl groups of polyols, amine groups, carboxyl groups, or the amount of water participating in the reaction [17]. The isocyanate index determines the degree of cross-linking and the content of urethane and isocyanate groups in the starting material, which in turn influences the reactivity, viscosity, molecular weight, and chemical composition of rebiopolyols. Foams with a higher INCO, containing more isocyanate groups, are characterized by a higher degree of cross-linking, which complicates chemical degradation but can lead to rebiopolyols with different physicochemical properties. Modesti et al. investigated the recyclability of polyisocyanurate wastes with a high isocyanate index by glycolysis and identified the optimal reaction conditions to obtain product suitable for reuse in the synthesis of new foams. The results showed that, despite the high thermal stability claimed for isocyanurates, under proper conditions the original, highly cross-linked structure of the polymer can be converted into a liquid, single-phase mixture terminated with hydroxyl groups [18].

Understanding the relationship between the isocyanate index of polyurethane foams and the properties of the resulting rebiopolyols is therefore crucial for designing effective chemical recycling processes and creating materials with controlled performance parameters. Research in this area makes a significant contribution to the development of modern polyurethane closed-loop technologies and supports the achievement of European goals related to raw materials and environmental policy. This study aims to systematically determine the fundamental properties of the repolyols and rebiopolyols obtained from polyurethane foams with varying isocyanate indices. Two groups of foams were investigated: one based on a petrochemical polyol and the other on a biopolyol derived from rapeseed oil. Within each group, the isocyanate index ranged from 0.5 to 1.1. To the best of our knowledge, such a comprehensive comparative analysis has not yet been reported in the literature, which highlights the novelty and originality of the presented research.

## 2. Experimental

### 2.1. Manufacturing of Rigid PUR Foams for Chemical Recycling

Rigid polyurethane foams were synthesized using a one-step process by combining an isocyanate component (PMDI) with a polyol or biopolyol, catalyst, surfactant, and water (a chemical blowing agent). The components were mixed mechanically for 4 s, after which the reactive mixture was poured into a mold to allow foam formation. The formulations of these rigid PUR foams are presented in Table 1. The reference foams (Ref1.1–Ref0.5) were obtained using petrochemical polyol RF-551 (produced by PCC Rokita) characterized by a hydroxyl value of 405 mg KOH/g. Biofoams were obtained using the biopolyol synthesized in the laboratories of Cracow University of Technology by epoxidation of rapeseed oil and the opening of oxirane rings with diethylene glycol. The hydroxyl value of the biopolyol was 240 mg KOH/g.

### 2.2. Chemolysis of PUR Foams and Biofoams

The chemical recycling of rigid polyurethane foams was performed via glycolysis, using diethylene glycol as a chemolysis agent and potassium hydroxide as the catalyst. Ground rigid PUR foams were subjected to a glycolysis reaction. Initially, diethylene glycol and the catalyst were heated to 180 °C under continuous stirring. Once the desired temperature was reached, the ground rigid PUR foam was gradually added in portions. The mass ratio of the ground PUR foam to diethylene glycol was 1:1. The foam was introduced portion-wise over a period of approximately 2 h. After the complete dissolution of the foam, the reaction mixture was maintained at 180 °C for an additional 30 min under constant stirring. The resulting rebiopolyols were used directly—without purification—for the synthesis of new polyurethane materials. The rebiopolyols were designated with the prefix “r”, followed by the name of the corresponding recycled foam.

### 2.3. Manufacturing of Rigid PUR Foams Based of Repolyol and Rebiopolyol

The resulting repolyols and rebiopolyols were used to produce rigid polyurethane foams in which 100% of the polyol was replaced with repolyol or rebiopolyol. The names of the new foams include information about the foam from which the repolyol or the rebiopolyol was obtained, along with the suffix “PU.” The foam production procedure was maintained as in the case of the initial foams intended for the chemolysis process, and the formulations are presented in Table 2.

### 2.4. Characterization of Repolyols and Rebiopolyols

The hydroxyl number (OHv) of the glycolysates was determined by titration using the pyridine method, following the PN-93/C-89052/03 standard [19]. The procedure involved the use of an acetylating reagent consisting of acetic anhydride and dimethylformamide mixed in specific volume ratios. A catalyst solution prepared by combining dimethylaminopyridine with dimethylformamide in defined proportions was employed, while a thymolphthalein solution in dimethylformamide served as an indicator.

The amine number (Amv) of the glycolysates was determined by titration according to the BN-69/6110–29 standard [20]. Approximately 0.3 g of the glycolysate sample was dissolved in a mixture of 40 cm^3^ of acetone and 10 cm^3^ of demineralized water. After dissolution, approximately five drops of bromocresol green indicator were added, and the solution was titrated under continuous stirring with 0.1 mol/dm^3^ hydrochloric acid until a yellow endpoint was reached. A blank test was performed following the same procedure, omitting the glycolysate sample.

The average molecular weight was determined by gel permeation chromatography (GPC) using an Azura® chromatograph manufactured by Knauer (Berlin, Germany). The chromatograph was equipped with thermostated columns and a refractometric detector. Tetrahydrofuran was used as the eluent at a flow rate of 1 cm^3^/min. The analysis was performed at 35 °C. The number average molecular weights (Mn) and weight average molecular weights (Mw) were determined based on the calibration for polystyrene standards with molecular weights of 410–20,500 g/mol.

The viscosity was measured using a Lamy Rheology CP-4000 device (Champagne-au-Mont d’Or, France) in a plate-to-plate configuration. Measurements were taken at a temperature of 25 °C and a rotation speed of 100 rpm.

The water content was determined using the Karl Fischer method in accordance with PN-81/C-04959 [21], using a TitroLine TA 05 plus device from SCHOTT Instruments GmbH (Meinz, Germany). The method is based on the reaction of Fischer’s reagent with water, and the equivalence point is determined by volumetric titration.

The chemical structure was analyzed using a Nicolet iS5 FTIR spectrometer (Thermo Fisher Scientific, Waltham, MA, USA). The device was equipped with an ATR i7D attachment with a diamond crystal. 

### 2.5. Characterization of Polyurethane Foams and Biofoams

The foaming process analysis was performed using a FOAMAT^®^ device manufactured by Format Messtechnik GmbH (Karlsruhe, Germany), according to ASTM D 7487-18 [22] and EN 14315-1 [23]. The device is equipped with a computer, a laboratory scale, a mechanical stirrer, an ultrasonic sensor, a foam pressure measurement device, a dielectric polarization sensor, and a thermocouple. During the foaming process, the following parameters are measured: temperature, rise pressure, dielectric polarization, weight loss, and foam height. Samples are prepared by mixing the polyol premix with isocyanate and pouring the mixture into a measuring tube. The thermocouple is then placed in a previously hollowed-out hole in the tube.

The apparent density of the foams was tested in accordance with ISO 845 [24]. Samples measuring approximately 20 × 20 × 5 cm were cut from the foam material obtained. Their length, width, and height were measured using a caliper. The weight of the tested samples was then measured on a technical scale. The volume of the sample was calculated as the product of the obtained values of length, width, and height. The apparent density was calculated as the arithmetic mean of the apparent density values for samples of three foams with identical formulations.

The morphology of cells was analyzed using a scanning electron microscope TM3000 (Hitachi, Tokyo, Japan). Foam samples of 1 × 1 × 1 cm before observation were covered with gold using a Polaron SC7640 duster (Quorum Technologies, Newhaven, UK). The sputtering process was carried out for 90 s at a current of 10 mA. Observations were carried out at an accelerating voltage of 15 keV.

The closed cell content was tested in accordance with ISO 4590 [25]. Samples measuring approximately 3 × 3 × 10 cm were cut from the material obtained. The length, width, and height of the samples were measured using a caliper. The closed cell content for a foam with a given formulation is obtained by calculating the arithmetic mean for four samples of the tested foam.

The thermal conductivity coefficient (λ) of the rigid polyurethane foams was determined using a Laser-Comp FOX 200 device manufactured by TA Instruments (New Castle, DE, USA). The temperature difference between the hot and the cold plates was 20 °C. The same sample as the one used in the apparent density test, measuring 20 × 20 × 5 cm, was utilized for the test. The device performs 8–12 series of measurements, on the basis of which the thermal conductivity coefficient is determined. The average thermal conductivity coefficient was calculated using the measurement results obtained for two samples of each foam.

The compressive strength was tested in accordance with ISO 844 [26] with a Zwick Z005 TH device (Zwick GmbH & Co., Ulm, Germany). The compressive strength was measured parallel and perpendicular to the direction of foam growth at a deformation of 10%. In such a test, eight cylindrical samples with a diameter and a height of approximately 4 cm are cut from the rigid polyurethane foams, four samples are cut parallel and four perpendicular to the foam growth direction. Then, using a caliper, the diameter and the height of each sample are accurately measured and entered into the computer program controlling the device. A sample is placed between the plates of the machine and compression begins.

## 3. Results and Discussion

**Figure 1:**
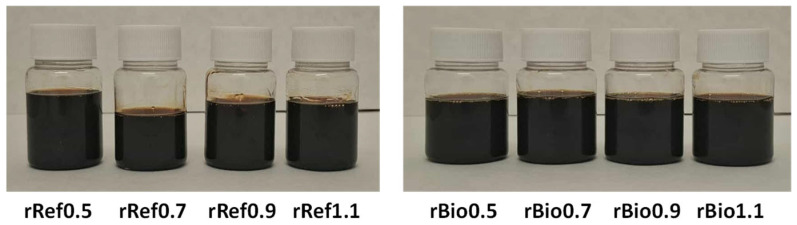
Photographs of the repolyol and rebiopolyol samples obtained via glycolysis of PUR and bioPUR foams.

**Figure 2:**
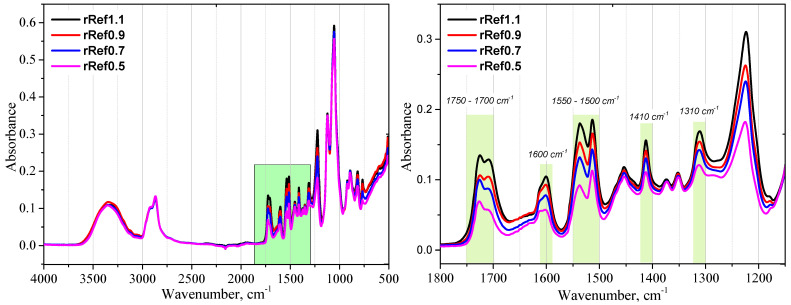
FTIR spectra of the repolyols.

The polyurethane (PUR) foams used in the glycolysis process had comparable apparent densities of approximately 90 kg/m^3^ and were ground to a particle size below 2 mm. The foams differed in chemical structure due to variations in the isocyanate index and the type of polyol used. Figure 1 presents real photographs of the obtained repolyols and rebiopolyols; the samples do not differ visually and all exhibit a uniformly dark coloration, typical for glycolysis products of polyurethane and biopolyurethane foams. To further investigate potential structural differences resulting from the chemical composition of the original foams, FTIR analysis was performed. The FTIR spectra of the repolyols are shown in Figure 2.

The FTIR spectra of the repolyols obtained by glycolysis of polyurethane foams show distinct differences in the intensity of the absorption bands located at 1750–1700 cm^−1^, 1600–1500 cm^−1^, 1410 cm^−1^, and 1310 cm^−1^, depending on the isocyanate index (NCO/OH) applied. A systematic decrease in the intensity of these bands was observed as the isocyanate index decreased from 1.1 to 0.5.

The bands in the 1750–1700 cm^−1^ region correspond to the C=O stretching vibrations of urethane (–NH–CO–O–) and urea (–NH–CO–NH–) carbonyl groups [6,10]. As the NCO content in the reaction mixture decreases, fewer urethane and urea linkages are formed, resulting in a reduced number of carbonyl-containing groups and, consequently, a lower band intensity in this region.

The band near 1600 cm^−1^ is mainly attributed to the C=C stretching vibrations in aromatic rings derived from MDI residues, often coupled with the N–H bending vibrations in urethane and urea linkages. A lower isocyanate index leads to a reduced incorporation of aromatic hard-segment fragments into the polymer network, which manifests as a decrease in the intensity of this band.

The region of 1550–1500 cm^−1^ includes the δN–H bending coupled with the νC–N stretching vibrations, typical of urethane and urea groups. The attenuation of these bands indicates a reduction in the number of nitrogen-containing linkages, directly resulting from the limited availability of –NCO groups during the foam synthesis [12,14].

The bands at 1410 cm^−1^ and 1310 cm^−1^ are also associated with the C–N stretching and N–H bending vibrations in urethane and urea structures. Their lower intensity at reduced NCO indices confirms a lower degree of cross-linking and a higher contribution of soft polyol segments.

Overall, the observed weakening of absorption bands in these regions of the FTIR spectra of the repolyols derived from foams with lower isocyanate indices can be directly correlated with the decrease in the number of urethane and urea linkages and the reduction in the aromatic hard-segment content in the parent polymer structure. This interpretation is consistent with previous literature, where the intensity of absorption bands in the 1750–1500 cm^−1^ region was shown to correlate with the hard-segment content and the degree of hydrogen bonding in polyurethane systems [27].

**Figure 3:**
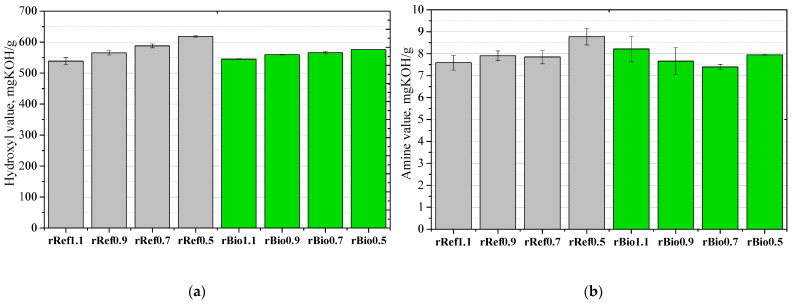
Correlation between hydroxyl value (**a**) and amine value (**b**) of repolyols and rebiopolyols with different isocyanate index of recycled PUR foams.

From the perspective of using polyurethane chemolysis products in new polyurethane systems, it is necessary to determine the hydroxyl number in order to develop formulations of new polyurethane materials. The results concerning the effect of the isocyanate index on the hydroxyl and amine number values for the repolyols and the rebiopolyols obtained from petrochemical foams and biofoams are presented in Figure 3.

In the case of hydroxyl number, it was observed that as the isocyanate index decreased, the hydroxyl number increased in both the petrochemical and the biopolyol-based foams. This effect was likely due to the presence of unreacted hydroxyl groups resulting from the isocyanate deficiency caused by the decreasing NCO index.

**Figure 4:**
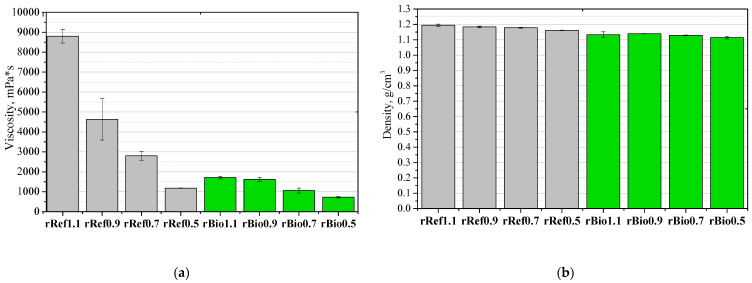
Correlation between viscosity (**a**) and density (**b**) of repolyols and rebiopolyols with different isocyanate index of recycled PUR foams.

The viscosity of repolyols and rebiopolyols is a key parameter determining their suitability for polyurethane processing, affecting the mixture’s rheology, component blending, and foaming behavior. Appropriate viscosity ensures an even distribution of reagents and a stable foam structure. Viscosity analysis allows for an assessment of the technological suitability and adjustment of formulation parameters (temperature, component ratios, mixing intensity) to obtain foams with consistent physicochemical and mechanical properties. Figure 4 shows the effect of the isocyanate index of the foams and the biofoams on the viscosity and the density of the resulting repolyols and rebiopolyols.

A strong correlation was observed: reducing the isocyanate index resulted in lower viscosities of the obtained repolyols and rebiopolyols. This effect is likely related to the degree of cross-linking of the polyurethane matrix. In the case of reference foams, the viscosity reduction is much more pronounced.

**Figure 5:**
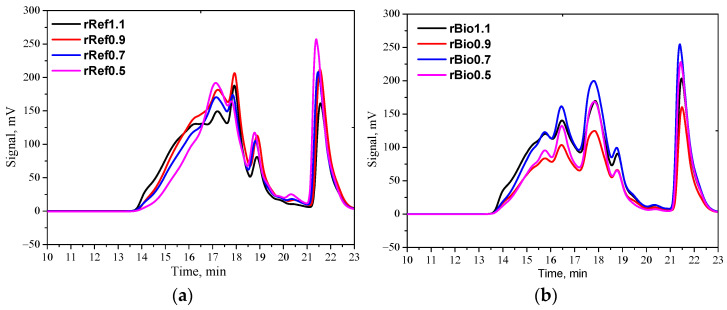
GPC chromatograms of repolyols (**a**) and rebiopolyols (**b**).

The viscosity values correlate with the average molar masses in the case of both the repolyols and the rebiopolyols. Figure 5 shows the GPC chromatograms of the repolyols (a) and the rebiopolyols (b) obtained from polyurethane foams and biofoams with different isocyanate indices. Table 3 shows their average molar masses and dispersity.

The observed reduction in the average molar mass of the repolyols with decreasing isocyanate index (from 1.1 to 0.5) is primarily due to the limited cross-linking potential at lower isocyanate contents. The isocyanate index determines the ratio of isocyanate to hydroxyl groups in the reaction; when the index is lower, fewer isocyanate groups participate in the formation of urethane bonds and networks, leading to shorter polyol chains in the repolyol and, therefore, a lower average molar mass.

**Figure 6:**
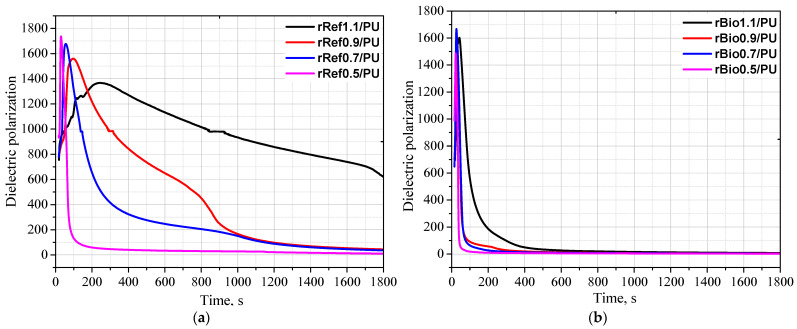

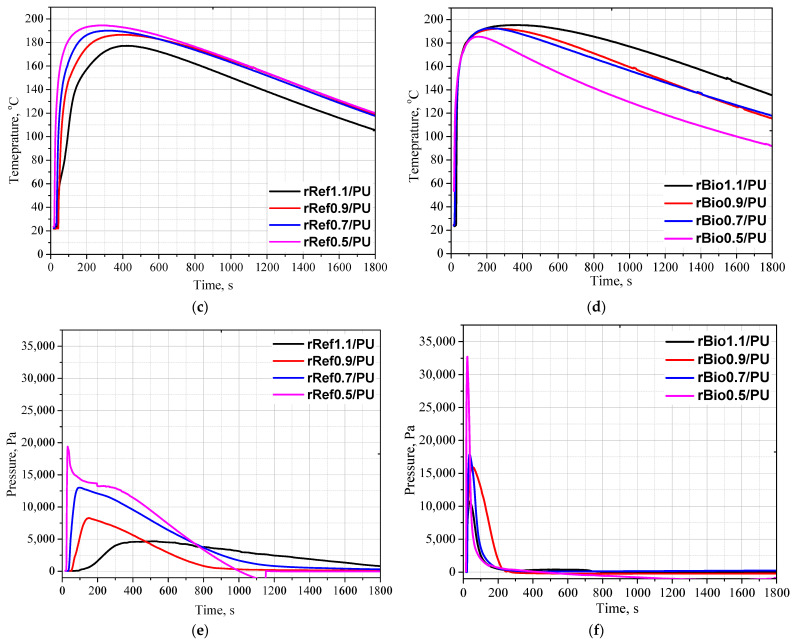
Influence of repolyols (**a**,**c**,**e**) and rebiopolyol (**b**,**d**,**f**) on the dielectric polarization, temperature and pressure during the foaming process.

**Figure 7:**
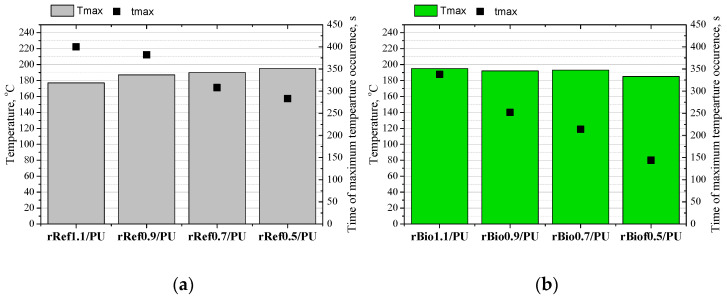
Maximum temperatures during the foaming process and the time needed to reach them for the systems modified with repolyols (**a**) and rebiopolyols (**b**).

The analysis of the impact of the repolyols and the rebiopolyols on the foaming process of polyurethane foams was conducted using a Foamat apparatus, which allows for a real-time analysis of changes in dielectric polarization, temperature, and pressure. The most important aspect of the research was the evaluation of the impact of new biocomponents on the foaming process, as it is during this phase that the foam’s cellular structure is formed, determining its subsequent physicochemical and mechanical properties. The study identified how the characteristics of the repolyols and the rebiopolyols—including viscosity, average molar mass, and chemical composition—affect the reactivity of the new polyurethane systems. Figure 6 shows the influence of the repolyols and the rebiopolyols on the changes in dielectric polarization, temperature and pressure during the foaming process, while Figure 7 shows the maximum temperatures during the foaming process and the time needed to reach them for the systems modified with the repolyols (a) and the rebiopolyols (b).

**Figure 8:**
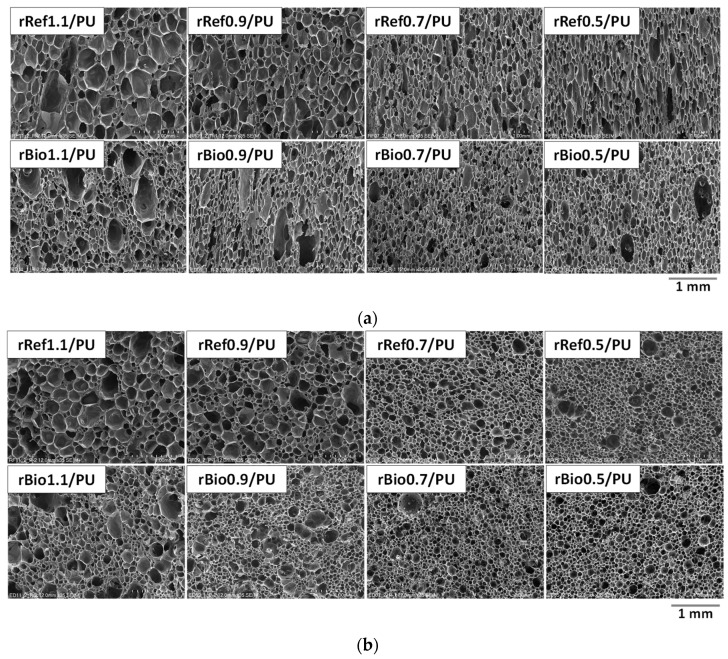
SEM micrographs of foams modified with repolyols and rebiopolyols taken in the direction parallel (**a**) and perpendicular (**b**) to the foam growth direction.

It was found that the isocyanate index of the foams that were used to derive the repolyols and the rebiopolyols has a significant impact on the foaming process. A decreasing isocyanate index increases the system’s reactivity, especially in the case of the repolyols. This effect is due to the fact that the modification of the repolyol system rRef1.1 significantly reduced reactivity, which may be related to the high viscosity of this repolyol. Lower viscosities of repolyols result in higher reactivity of polyurethane systems. It was observed that in the case of the systems modified with repolyols, the maximum temperature during the foaming process was higher and reached in a shorter time. In the systems modified with the rebiopolyols, the maximum temperature was achieved faster. However, as the isocyanate index of the source foam decreased, the maximum temperature during the foaming process decreased slightly. The reactivity of the system affects the cellular structure. Figure 8 shows SEM images of the polyurethane foams obtained.

**Figure 9:**
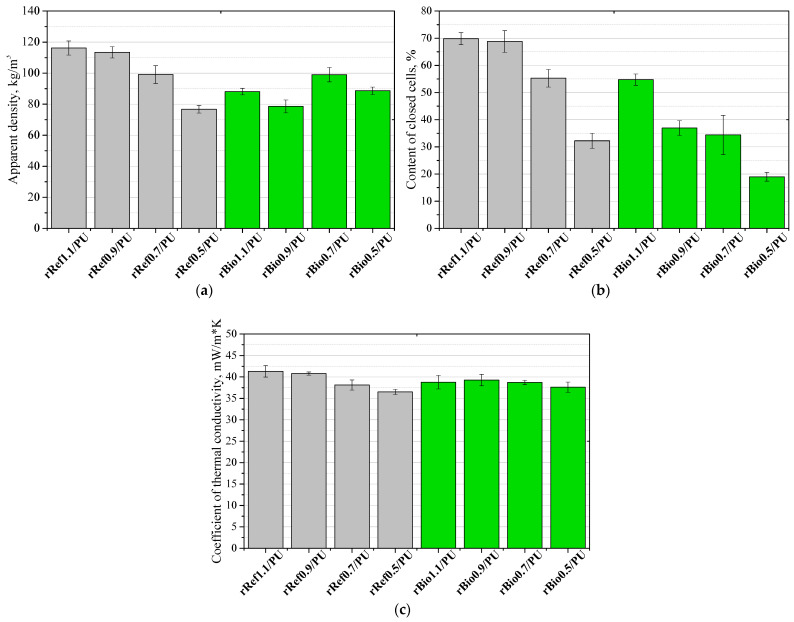
Effect of repolyols and rebiopolyols on apparent density (**a**), closed cell content (**b**) and thermal conductivity coefficient (**c**).

It can be clearly stated that the systems with lower reactivity are characterized by significantly larger cells both parallel and perpendicular to the foam growth direction. Gelation reactions take longer to complete at lower reactivity, thus giving cells more time to grow. Figure 9 shows the effect of the repolyols and the rebiopolyols on the apparent density (a), closed cell content (b) and thermal conductivity coefficient (c).

The apparent density of the resulting polyurethane foams ranged from 80 to 120 kg/m^3^. The addition of the repolyols and the rebiopolyols was found to affect the degree of cell openness. The degree of cell openness correlates with pressure changes during the foaming process. In the case of the repolyol-modified foams, the closed cell content values are higher, and for these systems, the pressure also increases during the foaming process compared to the systems modified with the rebiopolyols.

**Figure 10:**
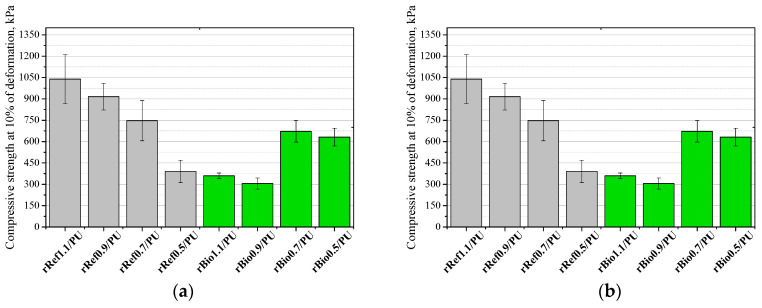
Compressive strength at 10% deformation measured in a direction parallel (**a**) and perpendicular (**b**) to the direction of foam growth.

A compressive strength analysis at 10% strain was performed in a direction parallel (Figure 10a) and perpendicular (Figure 10b) to the foam growth direction.

**Figure 11:**
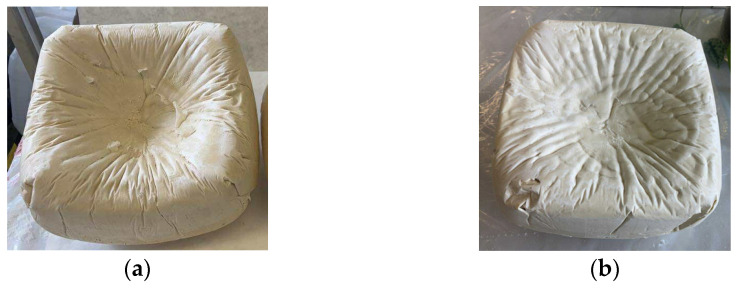
rBio1.1/PU (**a**) and rBio0.9/PU (**b**) foams with visible shrinkage.

The higher shrinkage tendency and reduced mechanical performance of rebiopolyol-based foams are primarily associated with structural instability occurring during foaming rather than with the isocyanate index alone. In the case of rebiopolyols obtained from foams prepared at a reduced isocyanate index, the decreased strength results directly from the limited crosslink density of the original material, as a lower NCO/OH ratio leads to the formation of a less densely crosslinked polyurethane network. During chemical recycling, such materials generate repolyols with a lower average molar mass and a higher content of low-molecular-weight degradation products, which limits the effective rebuilding of a stable polymer network. However, for rebiopolyol-modified foams, the lack of correlation between isocyanate index and mechanical properties is mainly governed by morphological instability during foaming, with noticeable shrinkage and cell structure disturbances (rBio1.1/PU and rBio0.9/PU) (Figure 11), which dominate the mechanical response and obscure purely chemical effects.

Shrinkage of polyurethane foams occurs when external pressure exceeds the mechanical strength of the cell walls, leading to their collapse and a reduction in foam volume. This phenomenon was less pronounced in the case of foams rBio0.7/PU and rBio0.5/PU foams, as these materials had higher apparent density and a greater degree of cell openness. Partially opening the cell structure allows for pressure equalization between the foam interior and the surroundings, limiting the effect of cell collapse. For the open-cell foams, the difference between the internal cell gas pressure within the foam and the external atmospheric pressure was zero and the dimensional stability problem is avoided [28].

## 4. Conclusions

Raw materials used in polyurethane synthesis offer considerable versatility and potential for designing diverse foam formulations. This study demonstrates that the isocyanate index significantly influences the properties of repolyols and rebiopolyols obtained via glycolysis. Foams with different isocyanate indexes were synthesized using both petrochemical polyols and biopolyol-based systems.

A reduction in the isocyanate index of the polyurethane foams that were used to obtain the repolyols and the rebiopolyols leads to a systematic decrease in the intensity of the bands that are characteristic for urethane and urea bonds in FTIR spectra. This is confirmed by a decrease in the number of carbonyl groups and a smaller share of hard segments in the polymer structure. A decrease in the isocyanate index results in an increase in the hydroxyl number of the repolyols and the rebiopolyols, which is a consequence of the presence of the unreacted hydroxyl groups following the deficiency of isocyanate groups in the parent foams.

As the isocyanate index decreases, the viscosity and average molar masses of the repolyols and the rebiopolyols decrease. This relationship is particularly pronounced for the repolyols derived from petrochemical foams. The cell structure of the foams modified with the repolyols and the rebiopolyols differs depending on the reactivity of the system—foams with lower reactivity exhibit larger cells, which is the result of longer gelation time and extended cell growth. Shrinkage was observed in the foams modified with the rebiopolyols, resulting from the predominance of external pressure over the mechanical strength of the cell walls. Such shrinkage was not observed in the foams with a higher apparent density and higher cell openness.

The results confirm that both the origin of the repolyols and the rebiopolyols, and the synthesis conditions of the parent foams (NCO index) strongly determine the course of the foaming reaction, the cell structure and the mechanical properties of the newly obtained polyurethane foams.

## Figures and Tables

**Table 1:** Formulations of rigid PUR foams prepared with different isocyanate indices.

Component	Ref1.1	Ref0.9	Ref0.7	Ref0.5	Bio1.1	Bio0.9	Bio0.7	Bio0.5
RF-551, g	100	100	100	100	0	0	0	0
Biopolyol, g	0	0	0	0	100	100	100	100
Polycat 9, g	1.5	1.5	1.5	1.5	1.5	1.5	1.5	1.5
L6988, g	1.5	1.5	1.5	1.5	1.5	1.5	1.5	1.5
Water, g	1	1	1	1	1	1	1	1
Index NCO	1.1	0.9	0.7	0.5	1.1	0.9	0.7	0.5

**Table 2:** Formulations of rigid PUR foams prepared based on repolyols and rebiopolyols.

Component	rRef1.1/PU	rRef0.9/PU	rRef0.7/PU	rRef0.5/PU	rBio1.1/PU	rBio0.9/PU	rBio0.7/PU	rBio0.5/PU
rRef1.1, g	100	0	0	0	0	0	0	0
rRef0.9, g	0	100	0	0	0	0	0	0
rRef0.7, g	0	0	100	0	0	0	0	0
rRef0.5, g	0	0	0	100	0	0	0	0
rBio1.1, g	0	0	0	0	100	0	0	0
rBio0.9, g	0	0	0	0	0	100	0	0
r.Bio0.7, g	0	0	0	0	0	0	100	0
rBio0.5, g	0	0	0	0	0	0	0	100
Polycat 9, g	0	0	0	0	0	0	0	0
L6988, g	1.5	1.5	1.5	1.5	1.5	1.5	1.5	1.5
Water, g	1	1	1	1	1	1	1	1
Index NCO	1.1	1.1	1.1	1.1	1.1	1.1	1.1	1.1

**Table 3:** Number average molar mass (Mn), weight average molar mass (Mw) and dispersity (D) of repolyols and rebiopolyols.

Symbol	Mn, g/mol	Mw, g/mol	D
rRef1.1	393	707	1.80
rRef0.9	358	634	1.77
rRef0.7	352	624	1.78
rRef0.5	318	556	1.75
rBio1.1	372	716	1.92
rBio0.9	352	676	1.92
rBio0.7	346	653	1.89
rBio0.5	332	638	1.93

## Data Availability

The original contributions presented in this study are included in the article. Further inquiries can be directed to the corresponding author.
